# Altered neurovascular coupling as measured by optical imaging: a biomarker for Alzheimer’s disease

**DOI:** 10.1038/s41598-017-13349-5

**Published:** 2017-10-10

**Authors:** Konstantin Kotliar, Christine Hauser, Marion Ortner, Claudia Muggenthaler, Janine Diehl-Schmid, Susanne Angermann, Alexander Hapfelmeier, Christoph Schmaderer, Timo Grimmer

**Affiliations:** 1 Department of Medical Engineering and Technomathematics, FH Aachen University of Applied Sciences, Campus Jülich, Heinrich-Mussmann-Str. 1, 52428, Jülich, Germany; 2 Department of Nephrology, Klinikum rechts der Isar, Technische Universität München, Ismaninger Str. 22, 81675 Munich, Germany; 3 Department of Psychiatry and Psychotherapy, Klinikum rechts der Isar, Technische Universität München, Ismaninger Str. 22, 81675 Munich, Germany; 4 Institute of Medical Statistics and Epidemiology, Klinikum rechts der Isar, Technische Universität München, Ismaninger Str. 22, 81675 Munich, Germany

## Abstract

Neurovascular coupling can be directly assessed by retinal vessel response to flickering light using optical imaging methods. The response is altered in a number of ocular and cardiovascular diseases. Whether it is altered in Alzheimer’s disease (AD) is investigated. Retinal vessel reaction to monochromatic flicker stimulation was examined by Dynamic Vessel Analyzer independent of the commercial software in elderly subjects: 15 patients with mild-to-moderate dementia due to AD (ADD); 24 patients with mild cognitive impairment due to AD (MCI); 15 cognitively healthy controls (HC). Retinal vessels in ADD showed a more emphasized and delayed reactive dilation as compared to HC. In MCI, these aspects still differed from those seen in ADD. Maximal arterial reaction was increased and dilation was delayed in ADD as compared to HC (p = 0.004 and p < 0.001) and to MCI (p = 0.058 and p = 0.004), respectively. Maximal venous reaction was increased in ADD as compared to HC (p = 0.001) and to MCI (p = 0.007), respectively. This finding suggests that retinal neuronal activity is either increased or feed-back loop of neurovascular coupling is damaged with differentiating alterations across the spectrum of AD. Thus, retinal vessel reaction to flicker stimulation is considered a promising non-invasive, widely available and easy-to-administer future biomarker for the diagnosis and monitoring of AD.

## Introduction

The characteristic histopathological features of Alzheimer’s disease (AD) include senile plaques and neurofibrillary tangles in conjunction with loss of neurons and synapses^1,2^. The major constituent of senile plaques is amyloid beta peptide (Aβ).

Mutations leading to an overproduction of Aβ are recognized as a major cause of aggregation of the peptide in early onset familial AD^3^. However, the reasons for β-amyloid deposition in late onset sporadic AD are less clear^4^. One hypothesis is that an impaired clearance of Aβ contributes to cerebral amyloid deposition^5^. This notion is strengthened by the finding that AD patients had identical Aβ production but decreased clearance rates relative to normal controls^6^. From animal studies it is known that molecules contained in the interstitial fluid (ISF) are cleared from the brain via different pathways. While ISF of white matter seems to be preferentially drained into the cerebrospinal fluid (CSF) directly, the interstitial fluid of gray matter appears to flow outward via perivascular spaces located alongside cerebral vessels and empty into cervical lymph nodes^7–11^. The latter drainage pathway could be impaired in late-onset AD due to impaired pulsatility of cerebral vessels. Consequently, amyloid may be less efficiently cleared from the brain and become deposited in the form of β-amyloid plaques. Consistent with this hypothesis, the progression of intracerebral amyloid deposition is predicted by the extent of white matter hyperintensities, a marker for small vessel disease in AD patients^12^.

Retinal vessels share anatomical and physiological features with cerebral small vessels as there is a common embryonic origin of the retina and the brain^13^. Retinal vessels can nowadays be visualized, quantified and monitored non-invasively and *in-vivo* with commercially-available retinal imaging technology.

The Dynamic Vessel Analyzer (DVA; IMEDOS Systems, Jena, Germany) measures retinal vascular dilatation in response to diffuse illuminance flicker. Several studies have reported reduced arterial and/or venous dilation in response to flicker in systemic and ocular diseases of vascular origin, for instance arterial hypertension^14^, diabetes mellitus^15,16^, metabolic syndrome^17^, glaucoma^18^, and age-related macular degeneration^19^. The reaction of retinal vessels to flicker light is influenced by neuro-vascular coupling^20,21^ and by retinal vascular function^14^. Since both aspects are assumed to be altered in AD^22,23^ a thorough examination of retinal vessel reaction to flicker using DVA in AD may contribute to the understanding of AD pathophysiology. For comprehensive reviews of the current evidence on the associations between neurovascular coupling and AD pathology please compare^24–26^.

The hemodynamic response as measured by Blood Oxygenation Level Dependent signals (BOLD) in functional MRI also depends on neuro-vascular coupling and appears to be reduced and delayed in patients with AD dementia^23,27^. We hypothesize that, similar to the BOLD response in AD, the temporal retinal vascular response to flicker light is diminished and delayed in comparison to healthy volunteers. Thus, we chose two main parameters for each vessel type to characterize these aspects of the temporal flicker curve: maximal vessel dilation and the reaction time of vessel response. Additionally, we aimed to investigate at what stage of the clinical spectrum of AD (healthy controls, mild cognitive impairment, mild AD dementia) the hypothesized alterations of retinal vascular response to flicker occur.

## Methods and Materials

### Ethics Statement

The study protocol was approved by the ethic committee of the Faculty of Medicine of the Technische Universität München (project number: 1118/14). All patients gave written informed consent, and all clinical investigations have been conducted in accordance with the principles of the Declaration of Helsinki, sixth revision.

### Sample size calculations

Due to the lack of studies on retinal vessel analysis in Alzheimer’s disease sample size for the study was estimated a priori for the main parameter: *mean maximal arterial dilation* based on previous studies on retinal vessel reaction in response to flicker in arterial hypertension^14^, diabetes mellitus^16^ and obesity^17^. In order to maintain the power 1-ß = 0.8 at α = 0.05 in the pairwise comparison of the subgroups we used a simplified formula for the sample size n in each group, while comparing means with two-sample t-test: n = 16*(σ/δ)^2^ ^28^ with σ = 1.9% as standard deviation of parameter values in each group and δ = 2, 0% as effect size. The estimation resulted in n = 14.

### Patient recruitment, inclusion and exclusion criteria

Three groups of participants were recruited:

- 15 patients, 72.9 ± 9.0 years old, with mild-to-moderate dementia due to probable AD fulfilling the standard diagnostic criteria^29^: *AD dementia group (ADD)*.
- 24 patients, 68.2 ± 9.2 years old, with mild cognitive impairment (MCI) due to AD^30^: *MCI group (MCI)*.
- 15 cognitively healthy control (HC) subjects, 66.4 ± 7.6 years old, with neither subjective nor objective cognitive impairment: *HC group (HC)*.

The MCI group was additionally divided into two subgroups according to positivity of AD biomarkers: MCI due to AD intermediate or high likelihood: *MCI-AD*, n = 13; MCI unlikely due to AD: *MCI-nonAD*, n = 11 using standard diagnostic criteria^30^.

Patients were recruited from the research outpatient Centre for Cognitive disorders at the Department of Psychiatry at the Technische Universität München. They had been referred for diagnostic evaluation of cognitive impairment by self-referral, general practitioners, neurologists, psychiatrists, or other institutions, and had undergone a standardized diagnostic procedure. HC subjects were mainly spouses of patients or volunteers recruited via word-of-mouth advertising. The standard diagnostic work-up included an interview with the patient and an informant as well as psychiatric, neurologic and physical examinations, neuropsychological evaluation including the Mini-Mental State examination (MMSE)^31^, and the Consortium to Establish a Registry for Alzheimer’s Disease Neuropsychological Assessment Battery (CERAD-NAB)^32^, a routine laboratory screen, and APOE genotyping. The severity of cognitive impairment was rated on the Clinical Dementia Rating scale (CDR)^33^; the sub-scores were used to calculate the CDR sum of boxes (CDR SOB). Cranial magnetic resonance imaging (MRI) was performed to assess structural brain abnormalities.

Patients incapable of providing written informed consent or those with major cardiac arrhythmias (for example atrial fibrillation), a known intolerance of tropicamide, glaucoma, distinct cataract, seizures, or amaurosis were excluded. Patients were not included in the study if they met diagnostic criteria for other neurological or psychiatric disorders, including Parkinson’s disease, normal pressure hydrocephalus, progressive nuclear palsy, or major depression. Patients were also excluded if they showed any major abnormalities on MRI, such as cerebral infarcts, extensive leukoencephalopathy, intracerebral aneurysm, or arteriovenous malformation. NINDS-AIREN criteria were used to exclude vascular dementia^34^. Furthermore, patients with other possible causes of cognitive impairment such as psychotropic medication (such as antidepressants, antipsychotics), substance misuse, or major abnormalities in routine blood testing were not enrolled.

All patients who met inclusion/ exclusion criteria were successfully examined and all datasets were usable for analyses.

### Retinal vessel analysis (RVA) assessment

Twenty minutes prior to the measurement, a drop of tropicamide (Mydriaticum Stulln: Pharma Stulln, Germany) was administered in the dominant eye of a subject, as assessed using the Dolman hole-in-the-card test^35^ to induce mydriasis. Image capturing for static vessel analysis was performed first, followed by dynamic vessel analysis recording with flicker stimulation. No intake of food or fluid was permitted 1 hour prior to the measurement. Participants also refrained from smoking and exercise during this time.

#### Static retinal vessel analysis

Static retinal vessel analysis was performed using the Static Vessel Analyzer (SVA, IMEDOS Systems, Jena, Germany) fitted with a Topcon NW200 infrared fundus camera (Topcon, Japan). 30° fundus images were captured and analyzed using standard software (VesselMap, IMEDOS Systems). We calculated the central retinal artery and vein equivalent (CRAE and CRVE, respectively) and arteriolar-to-venular ratio (AVR) as described elsewhere^36,37^.

#### Dynamic retinal vessel analysis

Retinal arterial and venous reaction to flicker stimulation was examined by Dynamic Vessel Analyzer (DVAlight, IMEDOS Systems) in all participants. We used standard 350 s measurement protocol described in detail elsewhere^17,19,38^. It included 3 consecutive periods of monochromatic flicker stimulation (530–600 nm, 12.5 Hz, 20 s). The standard locations for analysis were upper temporal retinal artery and vein 1–2 optic nerve head diameters away from the optic nerve head fence.

The quality of the DVA recordings were assessed semi-objectively using a cumulative scoring method (K. Kotliar, W. Smith, *et al*. submitted) with the following criteria: (a) Flicker evaluation is possible, (b) at least one evaluable flicker period (baseline/flicker pattern), (c) measuring points during flicker are consistent over the whole recording, (d) low noise, (e) almost no gaps in the recording. Each category was estimated subjectively by an experienced rater (KK) using the sub-scores 0 (“not true”), 0.5 (“partially true”), or 1 (“true”). These sub-scores were added resulting in the quality score of a recording ranging from 0 (“bad quality”) to 5 (“excellent quality”). Based on this assessment, only DVA recordings with score values ≥ 2.0 were included into further evaluation. If the quality of the DVA recording was lower (occurred in two participants) another vessel segment was measured.

Parameters of dynamic vascular response were assessed and analyzed independent of the commercial DVA software. A template with corresponding macros in a spreadsheet (Excel; Microsoft) was created in order to filter, process, and analyze the numerical data from the DVA as described previously^17,19^.

**Figure 1:**
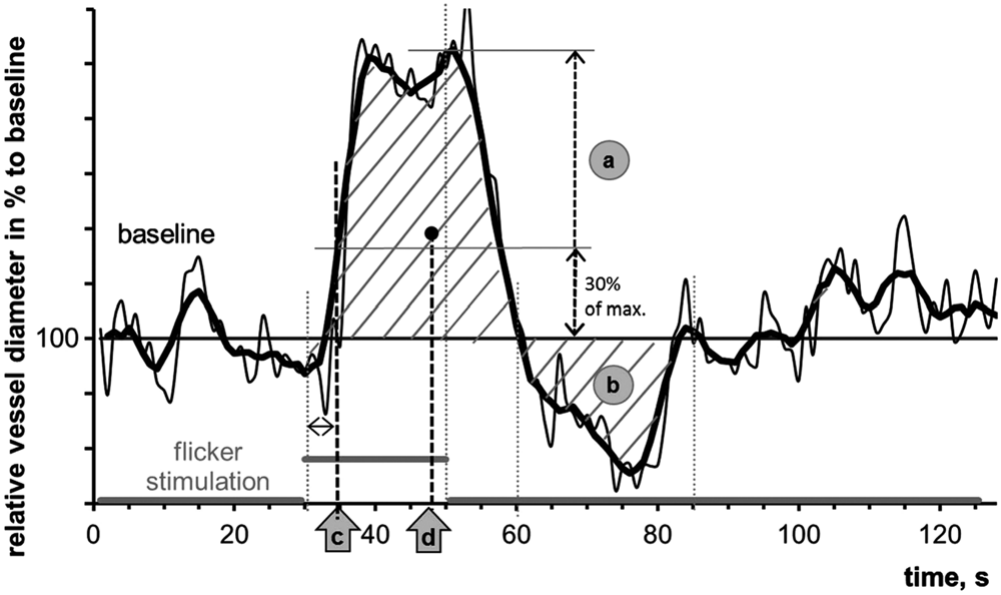
Some parameters of retinal vessel reaction to flicker reported in the study. Clarification of other parameters elsewhere^17^. The thin black line shows the median vessel diameter from the 3 flicker cycles. The superimposed solid black line illustrates the running median (4 s frame and the corresponding back shift) used to smooth the data. All vessel parameters were calculated using the values from this running median line (see detailed explanation in the text). The following parameters were derived from the flicker curve: (**a**) mean maximal dilation in response to flicker, [% to the baseline]; (**b**) area under the reaction curve after flicker cessation, [%×s]; (**c**) time to reach 30% of maximal dilation at the ascending slope, taking flicker initiation as 0, [s]; (**d**) time to reach the “center of gravity” of the area under the flicker curve over the baseline between the flicker initiation and the first baseline intersection after the peak dilation, [s].

In brief, absolute vessel diameter of measured arterial and venous segments was calculated individually as a median value during final 30 seconds before the first flickering. This parameter was measured in measuring units (MU) corresponding to µm in the Gullstrand’s eye^39^. Three single curves obtained during each flicker cycle in each subject and consisting of 30 s of baseline before the flicker application, 20 s of flicker application and 80 s thereafter were recalculated in % to their baseline values and averaged to one^17,19^. For each individual this averaged time course of relative vessel diameter changes was smoothed using the running median (4 s frame) and the corresponding back shift (Fig. 1). Parameters of dynamic retinal vessel reaction (Table 1) were calculated for each subject.

**Table 1:** Parameters of dynamic retinal vessel reaction to flickering light.

nr.	parameter	unit	description, explanation	link to Fig. 1
1	**mean maximal dilation in response to flicker**	% to baseline	is calculated as absolute maximum of the curve	a
2	time of maximal vessel dilation	s	takes flicker initiation as 0 s	
3	area under the reaction curve (AUC) after flicker cessation	%*s	is calculated between 10–40 s after the end of the stimulation. For the values under the 100%-line the area was negative	b
4	mean maximal constriction after flicker stimulation	% to baseline	(diameter decrease for veins) is calculated as absolute minimum of the curve. For the curves under the 100%-line the value was negative	
5	time of maximal vessel constriction	s	takes flicker initiation as 0 s	
6	arterial reactive magnitude	% to baseline	is calculated as a difference between mean maximal dilation and constriction	
7	AUC long term after flicker cessation	%*s	is calculated between 70–100 s after the end of the stimulation	
8	**time to reach 30% of maximal dilation at the ascending slope**	s	together with 9. a novel parameter, characterizing temporal shift of the flicker curve. Takes flicker initiation as 0 s	c
9	time to reach the “center of gravity” of AUC	s	implies “center of gravity” of the AUC over the baseline between the flicker initiation and the first baseline intersection after the peak dilation. Takes flicker initiation as 0 s	d

Legend to Table 1: Parameters calculated from averaged smoothed individual time courses. Parameters of the primary analysis are accentuated bold. AUC: area under the reaction curve.

Representative intermediate time courses of vessel diameter changes in each group were plotted as a median of all smoothed individual temporal responses of the group^17,19^. The median time courses show the dynamic behavior of vessel diameter of a group with the following limitation: some introduced statistical parameters may not exactly match a corresponding value on the curve because of the method of calculation.

### Statistical analysis

Descriptive statistics of non-normally distributed data are given by median and interquartile range (IQR = 1^st^ to 3^rd^ quartile). Corresponding pairwise group comparisons were performed by Mann-Whitney-U-tests. The distribution of categorical data was presented by absolute and relative frequencies and compared between groups by means of a χ^2^-test.

We controlled the two parameters, a-priori defined by our main hypothesis (primary analysis), for multiple comparisons with the Holm-Bonferroni procedure^40^:

- reaction magnitude using *mean maximal dilation in response to flicker;*
- reaction delay using *time to reach 30% of maximal dilation at the ascending slope*.

Comparisons of other parameters were carried out exploratively without correction for multiple comparisons in order to show tendencies of the different vessel behavior in the groups and to suggest additional candidates for biomarkers characterizing retinal vascular alterations in AD dementia. Spearman correlation coefficients were calculated to reveal associations between chosen biometric and retinal vessel parameters. ROC-analysis was performed to show the detection quality of parameters. All statistical hypothesis testing was conducted on two-sided 5% significance levels.

Statistical analysis was performed using IBM SPSS v. 21 (IBM, Armonk, USA) as well as Primer of Biostatistics, v.4.03 by Glantz^41^.

### Data availability statement

The datasets generated during and/or analyzed during the current study are available from the corresponding author on reasonable request. However, due to the nature of pseudonymized patient data, a material transfer agreement is required to meet ethical standards and data privacy laws of Germany.

## Results

In general, our groups showed highly significant differences in established psychometric and clinical scales related to dementia, especially AD dementia (Table 2). Although the groups were of similar age, the ADD was slightly older (p = 0.024) than both other groups. Blood pressure levels were similar and rather hypertensive, frequency of diabetes, hypertension and smokers were similar distributed across groups (Table 2).

**Table 2:** Values of biometric and systemic parameters in the cohort.

parameter/group	ADD (n = 15)	MCI (n = 24)	HC (n = 16)	exact p-values		
	3	2	1	1–3	2–3	2–1
Sex [male:female]	6:9 (40%)	10:14 (42%)	6:10 (38%)	0.922	0.943	0.838
Age, [years]	73.7 (67.1–79.8)	69.2 (63.5–73.2)	68.6 (60.2–71.1)	*0.024*	*0.033*	0.946
Z-Score MMSE	−5.0 (−15.8 – −3.2)	−2.4 (−4.3 – −1.0)	0.0 (−1.3–0.5)	<*0.001*	*0.001*	*<0.001*
CERAD NAB sum score	45.0 (41.0–64.0)	68.5 (60.5–76.5)	86.0 (79.0–92.5)	*<0.001*	*0.001*	*<0.001*
CDR global	1.0 (1.0–1.0)	0.5 (0.5–0.5)	0.0 (0.0–0.0)	*<0.001*	*<0.001*	*<0.001*
CDR-SOB	5.0 (4.5–6.0)	2.5 (1.5–3.3)	0.0 (0.0–0.0)	*<0.001*	*<0.001*	*<0.001*
Dominant eye [right:left]	5:10 (33%)	12:12 (50%)	9:7 (56%)	0.281	0.399	0.754
Awake for [hours]	6.3 (5.8–7.3)	6.8 (5.5–7.5)	6.5 (5.6–7.3)	0.800	0.516	0.563
Duration of sleep last night, [hours]	9.0 (8.5–9.5)	8.5 (7.0–9.0)	8.0 (7.5–8.5)	*0.011*	0.089	0.437
Caffeine	11 (73%)	19 (79%)	16 (100.0%)	0.216	0.765	0.279
Nicotine	1 (7%)	4 (17%)	1 (6%)	0.984	0.618	0.594
Diabetes mellitus	0 (0%)	2 (8%)	0 (0%)	1.0	0.508	0.508
Arterial Hypertension	11 (69%)	10 (42%)	6 (40%)	0.156	0.117	1.0
Systolic blood pressure [mmHg]	135.0 (124.3–143.0)	133.0 (121.0–144.5)	133.5 (125.5–142.0)	0.851	0.797	0.944
Diastolic blood pressure [mmHg]	83.5 (81.0–87.3)	85.0 (77.3–89.0)	84.0 (78.8–86.3)	0.668	0.620	0.910

Legend to Table 2: Absolute (relative) frequency or median (1^st^ quartile – 3^rd^ quartile) where appropriate. Significance for categorical (χ^2^_-_test) and continuous variables (Mann-Whitney-U-test). Exact p-values reported without correction for multiple comparisons. P-values < 0.05 are accentuated with italic. ADD: Alzheimer’s disease dementia, HC: cognitively healthy controls. MCI: mild cognitive impairment. MMSE: Mini-Mental state examination; CERAD NAB: Consortium to Establish a Registry for Alzheimer’s Disease Neuropsychological Assessment Battery; CDR-SOB: Clinical dementia rating sum of boxes.

Values of retinal vessel analysis parameters in the investigated groups are represented in Table 3.

**Table 3:** Values of parameters of static retinal vessel analysis and retinal vessel reaction to flickering light.

parameter/group	ADD (n = 15)	MCI (n = 24)	HC (n = 16)	exact p-values		
	3	2	1	1–3	2–3	2–1
DVA data quality, [subjective score 1.0–5.0]	4.0 (2.8–5.0)	4.0 (3.0–5.0)	4.5 (4.0–5.0)	0,121	0,295	0,424
central retinal arterial equivalent, [MU]	161.8 (139.1–168.9)	163.4 (145.7–174.9)	160.6 (152.1–166.2)	0.458	0.397	0.722
central retinal venous equivalent, [MU]	197.6 (185.1–211.8)	204.6 (193.7–217.0)	205.6 (196.8–212.2)	0.155	0.190	0.699
arterio-venous ratio, AVR	0.77 (0.76–0.84)	0.77 (0.71–0.86)	0.78 (0.75–0.81)	0.583	0.795	0.769
arterial diameter, [MU]	111.7 (99.6–133.7)	108.5 (103.9–112.5)	107.6 (101.9–119.1)	0.740	0.700	0.881
**mean maximal arterial dilation**, [% baseline]	6.6 (3.9–8.7)	3.8 (2.0–5.4)	2.7 (1.9–3.5)	*0.004 (<0.02)*	0.058	0.126
time of maximal arterial dilation, [s]	21.5 (16.5–24.3)	14.5 (11.5–19.8)	17.5 (13.8–20.3)	0.101	*0.015*	0.503
mean maximal arterial constriction, [% baseline]	−1.3 (−2.3 – −0.9)	−1.9 (−2.3 – −1.1)	−1.2 (−1.7 – −0.8)	0.401	0.466	0.058
arterial AUC after flicker cessation, [%*s]	15.7 (−8.7–43.9)	−19.7 (−39.0–4.8)	−6.0 (−19.4–2.9)	0.101	*0.017*	0.202
time of maximal arterial constriction, [s]	64.0 (35.5–76.3)	49.0 (37.8–60.3)	52.5 (40.5–75.0)	0.922	0.484	0.633
arterial reactive magnitude, [MU]	8.1 (5.4–9.7)	5.3 (3.7–7.2)	3.7 (3.1–4.4)	*0.007*	0.138	*0.034*
**arterial time to reach 30% of max. dilation**, [s]	7.0 (6.0–11.0)	5.0 (3.0–7.0)	5.0 (3.0–6.0)	*<0.001 (<0.01)*	*0.004 (<0.02)*	0.853
arterial time of center of gravity at flicker, [s]	21.4 (19.8–27.7)	17.7 (16.1–19.1)	16.7 (15.4–17.8)	*0.002*	*0.005*	0.267
venous diameter, [MU]	140.5 (125.7–150.7)	133.2 (120.5–145.2)	143.5 (135.7–155.9)	0.232	0.658	0.101
**mean maximal venous dilation**, [% baseline]	5.4 (5.2–6.6)	4.7 (2.9–5.2)	3.7 (2.9–4.7)	*0.001 (<0.01)*	*0.007 (<0.02)*	0.557
time of maximal venous dilation, [s]	23.0 (20.5–24.0)	20.5 (15.0–23.0)	21.0 (18.3–23.3)	0.446	0.123	0.521
mean maximal venous constriction, [% baseline]	−1.2 (−1.5 – −0.7)	−1.1 (−1.7 – −0.5)	−1.0 (−1.6 – −0.5)	0.892	0.875	0.967
time of maximal venous constriction, [s]	68.0 (53.0–84.3)	71.5 (49.5–81.0)	65.5 (48.5–75.5)	0.830	0.449	0.967
venous AUC after flicker cessation, [%*s]	14.0 (5.8–29.8)	11.8 (−6.6–25.0)	11.3 (−1.9–25.8)	0.318	0.212	0.774
**venous time to reach 30% of max. dilation**, [s]	7.0 (6.0–8.0)	7.0 (5.0–9.0)	6.0 (5.0–8.3)	0.232	0.700	0.404
venous time of center of gravity at flicker, [s]	22.2 (20.6–24.3)	20.9 (18.9–23.3)	21.4 (19.0–27.8)	0.740	0.202	0.576

Legend to Table 3: median (1^st^ quartile – 3^rd^ quartile), significance with Mann-Whitney-U-test. Exact p-values reported without correction for multiple comparisons. P-values < 0.05 are accentuated with italic. Parameters of the primary analysis are accentuated bold. For these parameters significant p-values after manual correction for multiple comparisons are shown in brackets. ADD: Alzheimer’s disease dementia group; HC: cognitively healthy controls; MCI: mild cognitive impairment.

Static retinal vessel parameters (CRAE, CRVE and AVR) did not show any remarkable differences between the groups (Table 3). A slight decrease of retinal venous equivalent was observed gradually with severity of cognitive impairment in MCI and ADD. AVR as an established clinical marker of microvascular dysfunction was reduced compared to normal values reported elsewhere^36,42^ and was similar across groups.

**Figure 2:**
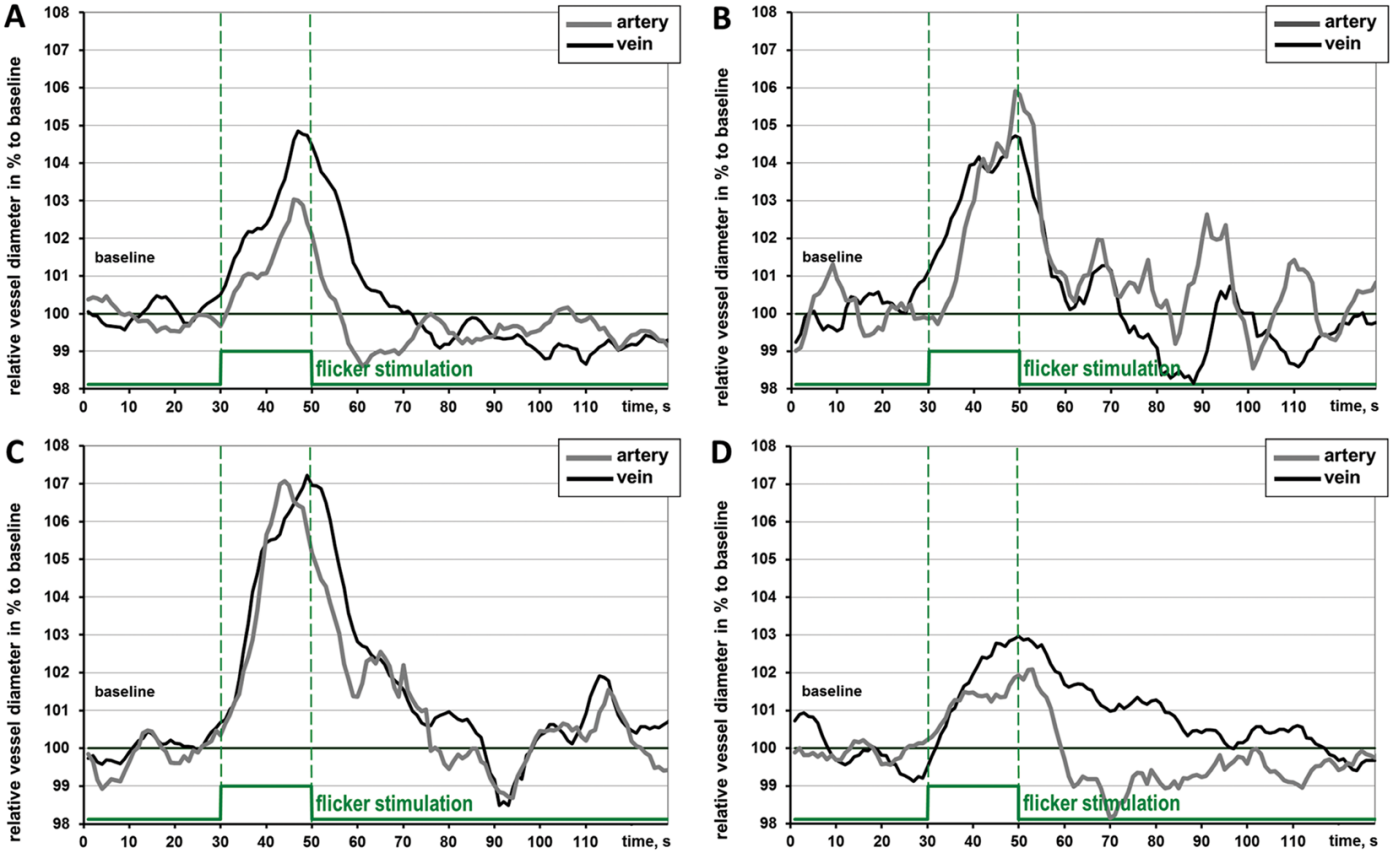
Characteristic examples of individual smoothed averaged retinal arterial and venous reactions to flickering light: (**A)** healthy control; (**B**) MCI-nonAD; (**C**) ADD; (**D**) MCI-AD.

The quality of dynamic vessel measurements was high enough to enable a qualitative data analysis and was well comparable in the groups amounting in average to at least 4.0 from 5.0 (Table 3). In most subjects fast vessel dilation compared to baseline was observed. Characteristic examples of individual retinal arterial and venous reactions to flickering light of all groups are shown in Fig. 2.

**Figure 3:**
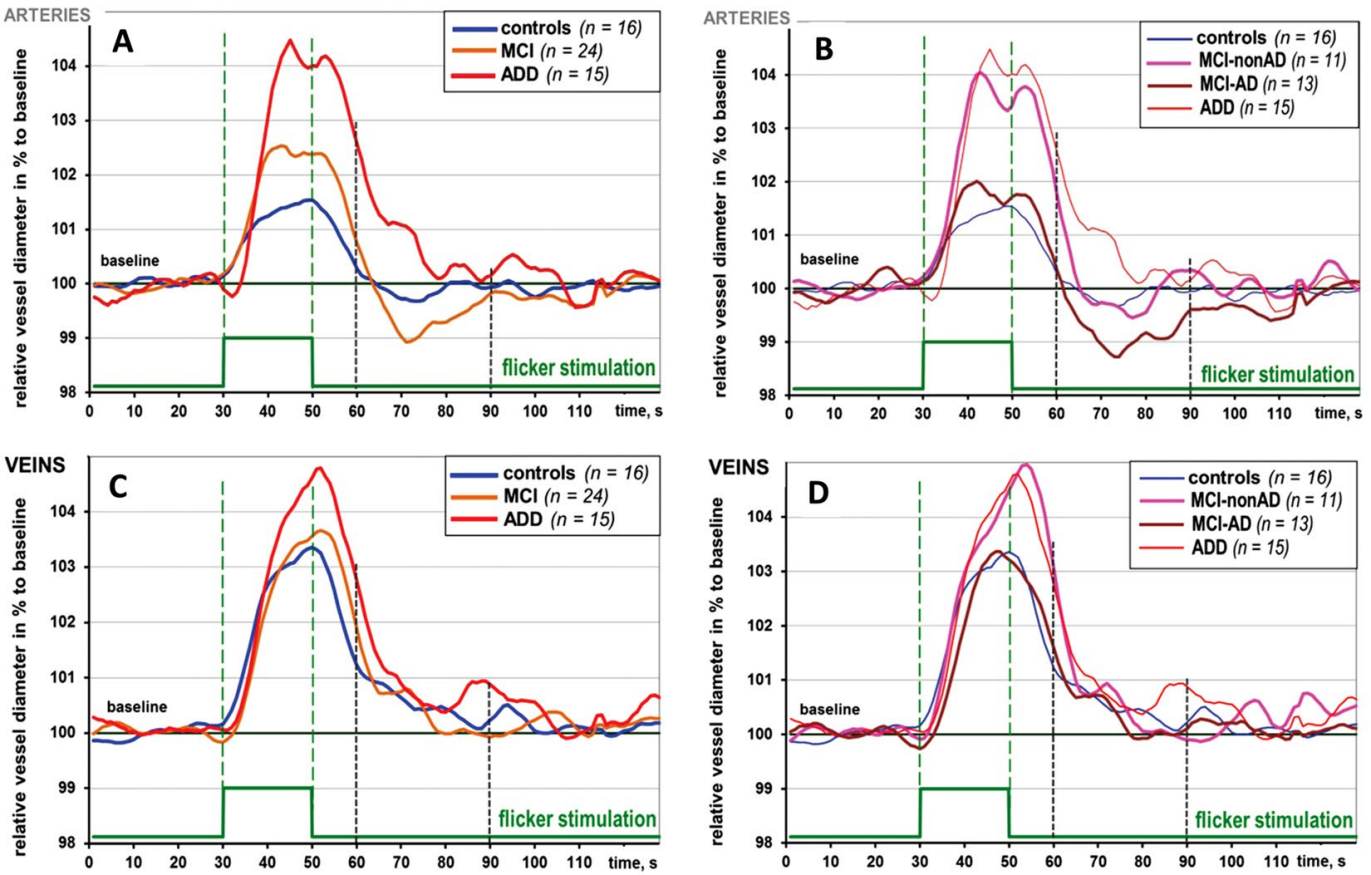
Arterial and venous reaction in the groups: relative vessel diameter changes in % to the baseline during flicker stimulation cycle (monochromatic, 12.5 Hz, 20 s). (**A**,**C**) MCI group uniform: (**B**,**D**) MCI group divided in two subgroups: MCI-nonAD and MCI-AD. Grey vertical stripes show the time interval 10–40 s after flicker cessation where arterial constriction and an emphasized decrease of venous diameter are expected in healthy volunteers.

In ADD both arterial and venous dilative responses increased immensely in comparison to the control group. The arterial reaction to flicker was delayed and lost the peculiar shape of a normal response reported elsewhere^17,20^ with its prompt emphasized constriction following the dilation phase (Figs 2A,C and 3A,C).

**Figure 4:**
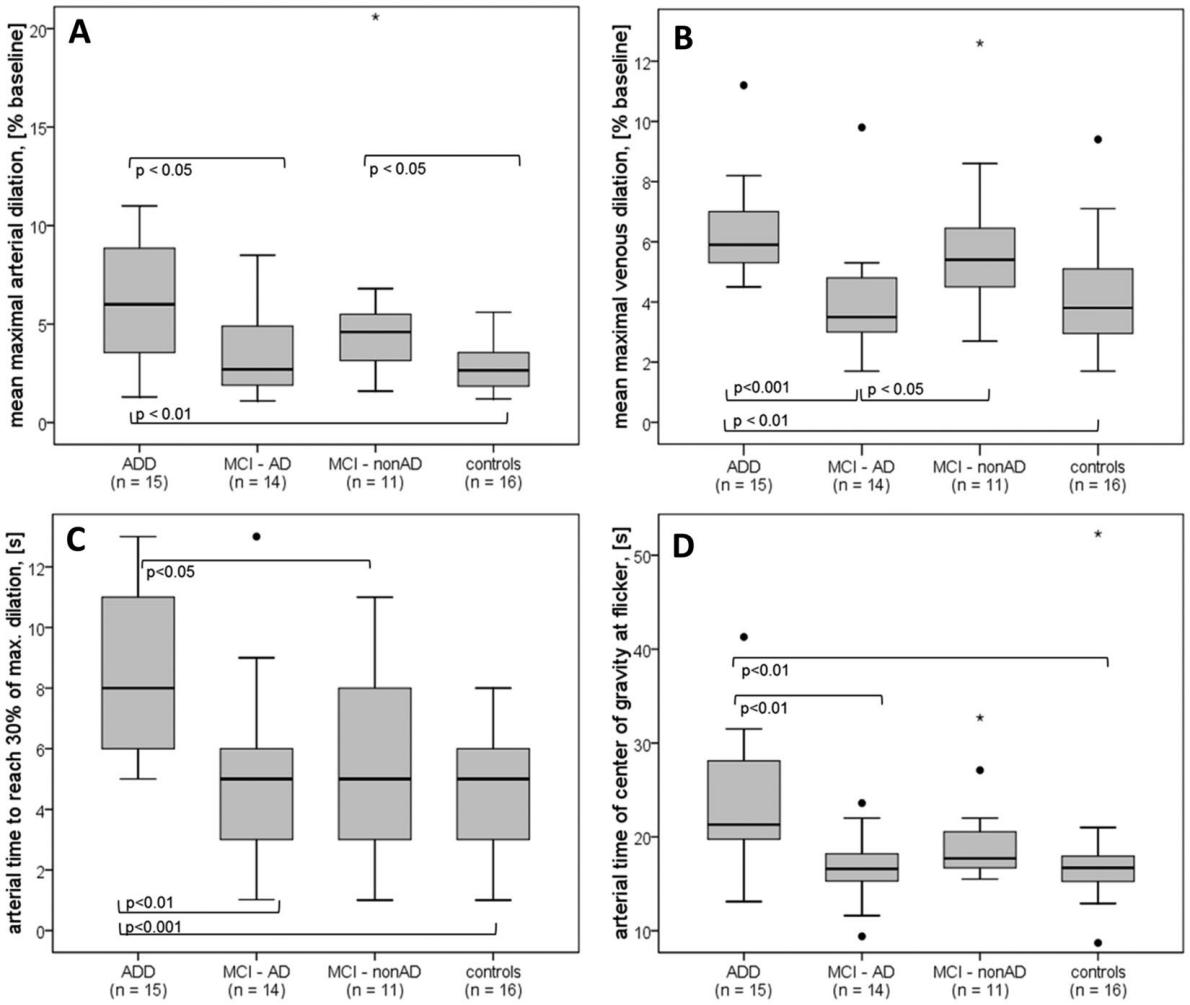
Maximal arterial and venous dilation (**A**,**B**) and latency parameters (**C**,**D**) in groups with MCI group divided in two subgroups: hypotheses testing with Mann-Whitney-U-Test. Exact p-values reported without correction for multiple comparisons. ^•^,*Suspected and qualified outliers respectively. Note an increased and delayed retinal vessel response in ADD

Retinal arteries in AD dementia showed more emphasized dilation in response to flicker in comparison both to HC and to MCI (p = 0.004 and p = 0.058 respectively; after correction for multiple testing: p < 0.02 and n.s., respectively. Table 3: “mean maximal arterial dilation”, Figs 3A and 4A). Retinal veins in AD dementia also showed more emphasized dilation compared to HC and to MCI (p = 0.001 and p = 0.007, respectively; after correction for multiple testing: p < 0.01 and p < 0.02, respectively. Table 3: “mean maximal venous dilation”, Figs 3C and 4B).

Arterial response in ADD was significantly delayed as compared to HC (p < 0.001; p < 0.01 after correction; Table 3: “arterial time to reach 30% of max. dilation”, Fig. 3A) and to MCI (p = 0.004; p < 0.02 after correction; Table 3, Fig. 3A). Interestingly, the time to reach 30% of the maximum response was quite similar in arteries and in veins in ADD (Table 3). Moreover, after the cessation of the flicker, retinal arteries tended not to constrict abruptly and become narrower than at baseline as reported in healthy volunteers^20,43^ but rather slowly change their diameter similar to the venous response in healthy subjects (Figs 2A,C and 3A,C).

Generally, the arterial flicker response curve differed in MCI as compared to HC. The reactive magnitude was significantly higher in MCI and the constriction was more emphasized (Table 3, Fig. 3A). Parameters in the Table 3 as well as in Fig. 3 show further details of retinal vessel response to flicker in the investigated groups.

As MCI was divided in two subgroups according to biomarkers of AD, the following results were observed in arteries and in veins (Fig. 3B,D): arterial response was increased in MCI-nonAD vs. HC (p = 0.03, Fig. 3B, Fig. 4A). Both arterial and venous responses were decreased in MCI-AD as compared to ADD (p = 0.041 and p < 0.001 correspondingly: Figs 3B,D and 4A,B). In addition, the venous response was reduced in MCI-AD vs. MCI-nonAD (p = 0.041, Figs 3D and 4B).

Arterial flicker response was significantly delayed in ADD vs. both MCI-AD and MCI-nonAD and HC (p = 0.007, p = 0.033 and p < 0.001 respectively, not corrected; Figs 3B and 4C). A similar effect was shown with another new latency parameter named the center of gravity (Table 2, Fig. 4D). Venous reaction in MCI-nonAD was similar to ADD, whereas venous reaction in MCI-AD was similar to HC (Figs 3D and 4B).

As to the regulation of arterial tone following the flicker stimulation phase, the shape of the arterial reaction after flicker cessation with its emphasized constriction was significantly different in MCI-AD as compared to HC (p = 0.032, “arterial AUC after flicker cessation” Fig. 3B) and to ADD (p = 0.001).

**Figure 5:**
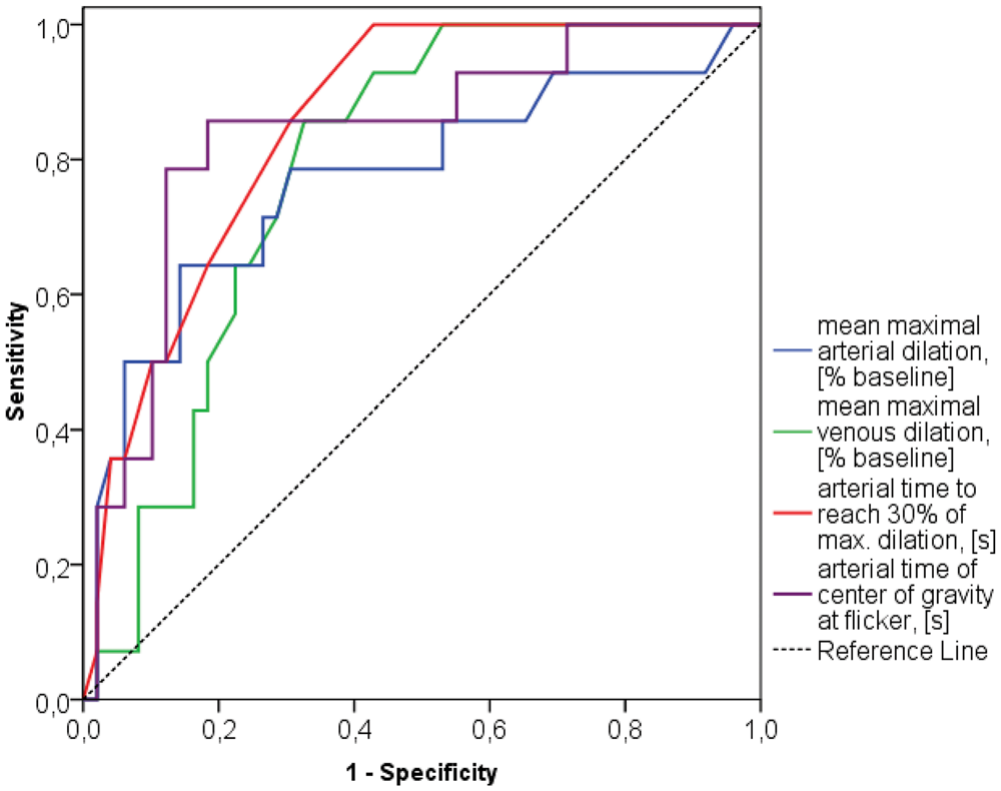
ROC-curves to reveal ADD for some parameters of the study [AUC (95% CI)]: mean maximal arterial dilation: AUC = 0.770 (0.613; 0.927), p = 0.003; mean maximal venous dilation: AUC = 0.786 (0.674; 0.898), p = 0.002; arterial time to reach 30% of max. dilation: AUC = 0.853 (0.758; 0.947), p < 0.001; arterial time of center of gravity at flicker: AUC =  0.837 (0.714; 0.959), p < 0.001.

ROC-curves show good prediction ability for the latency parameters in retinal arteries (AUC > 0.8) and useful prediction ability (AUC >  0.7) for the dilation parameters for retinal arteries and veins (Fig. 5)

Correlation between the parameter duration of sleep last night and mean maximal arterial dilation was not significant (p = 0.33): r = −0.24 (HC); r = −0.077 (ADD). Correlation between the same parameter and “time to reach 30% dilation” was not significant for HC (r = 0.292; p = 0.24): and moderately significant for ADD (p =  0.027; r = 0.550).

## Discussion

Although studies on retinal vessel analysis in AD have been done before^22^, (Golzan *et al*. AAIC 2014), to the best of our knowledge, this is the first study evaluating in detail the usefulness of retinal vessel flicker response parameters as possible biomarkers for the diagnosis of AD.

We are able to show that retinal vascular dilatator response to flickering light is unexpectedly emphasized in ADD in comparison to cognitively healthy controls and to MCI. Retinal arterial flicker response overall was significantly delayed in ADD in comparison to HC and to MCI. A small delay of venous response in ADD and MCI vs. HC showed a tendency that was, however, not significant.

Hence we can suggest that neurovascular coupling of retinal vessels in AD is altered in a very peculiar manner, especially in retinal arteries: The time course of retinal arterial reaction to flicker in ADD differs from the corresponding healthy pattern. Arteries in ADD dilate more with an emphasized delay and slowly reduce their diameter after flicker cessation similar to venous behavior observed in healthy controls.

The reason for the arterial upregulation could be damage of the feed-back loop of the regulation capacity at neurovascular coupling. Apparently, retinal neurons in AD work hard in order to process a visual stimulus of flickering light. They obtain an appropriate blood supply by retinal vessel dilation because of neurovascular coupling; however, compensatory mechanisms, the counterparts of the regulation responsible for the constriction do not function properly and allow the vessels to increasingly dilate. Another simpler but bold explanation of the observed upregulation in arteries: the neurovascular coupling is still intact in AD. It should reflect the rate of neuronal activity in this region. A higher activity means higher blood supply and stronger arterial dilation. A much emphasized dilation in ADD patients would then be a manifestation of the high activity of retinal neurons, which characterize this stage of the disease. An abnormally high neuronal activity in AD was reported elsewhere^44^. Further research of vascular dynamic function in AD should reveal the exact reasons for the upregulating behavior of retinal vessels in this pathology.

Results of ROC-analysis (Fig. 5) show that suggested magnitude and latency parameters of retinal vessel response to flicker represent good candidates for specific and sensitive biomarkers of AD and they should be investigated further for this purpose. Presumably, a combination of different parameters of retinal vessel behavior would be promising for characterizing and monitoring of AD.

In MCI-AD, arterial responses decreased and showed an emphasized constriction after flicker cessation (Fig. 2). Our previous studies showed that such a shape of the arterial response is rather associated with a healthy reaction and an improvement of retinal arterial function^14,42^. In any case, this change of MCI-AD towards HC and ADD reflects alterations in the vascular regulation in MCI-AD. The difference in the corresponding parameter *“AUC after flicker cessation between 10–40* *seconds after the end of the flicker stimulation”* between HC and MCI-AD and between MCI-AD and ADD was significant (p < 0.05 and p < 0.001 correspondingly, Fig. 3)

The correct definition of latency parameters was one of the strengths of our study. In the current study, the parameter time of maximal arterial dilation did not show differences between the groups (Table 3). This finding can be explained with a known high interindividual variability of this parameter in the population: in healthy subjects^17,42^, but also in some of our subgroups (Fig. 3) the reaction curve possesses two consequent peaks. The absolute maximum of the curve can be found on the first or on the second peak. Finally we wanted to describe quantitatively the retardation of the dilative reaction to flicker. To this end, we proposed two novel latency parameters: a primary one showing the initial delay in the beginning of the flicker response, *time to reach 30% of maximal dilation at the ascending slope*, and a secondary one, *time to reach the “center of gravity” of the area under the flicker curve*, reflecting how the whole dilation process is temporally shifted (Fig. 1). Both parameters show clearly the delay of arterial reaction in ADD in the present study and a tendency of venous delay in MCI and ADD towards HC.

The study design is a further strength of our study: we compared ADD and MCI patients with a HC group of similar age and vascular status who lacked subjective and objective cognitive impairment. Blood pressure values (Table 2) and AVR-values (Table 3) were well comparable across groups. Blood pressure level was increased and AVR level was decreased in all the groups in comparison to the standard value range. According to the experimental paradigm the results of the study allow us to affirm that the changes in retinal vessel response to flicker are primarily due to factors related to AD and not to the effects of aging or possibly related vascular dysfunction.

Mroczkowska *et al*. showed in a short report that maximum retinal arterial reaction to flicker occurred later in the first and the third of three flicker cycles in AD as compared to HC earlier in the second^22^. This result allowed the authors to report retinal vascular dysfunction in AD. The delay of maximal arterial dysfunction in AD would be compatible with our finding of significant delayed arterial response in ADD as compared to HC.

Golzan *et al*. (AAIC 2014) presented data of a reduced retinal arterial and venous flicker response in a small ADD cohort. The discrepancy of these results to the results of the present study is explainable. Presumably, the authors studied less severe stages of AD and compared it with younger, healthier volunteers regarding cardiovascular risk factors. In this case the reaction in ADD would be weaker and similar to the reaction of MCI-AD in our study (Fig. 3), whereas younger and healthier controls would show a more emphasized dilative reaction^42,45^. As a result, one would show a reduced reaction in ADD towards HC. We want to emphasize again that the experimental paradigm and the findings of the present study are more sensible from a clinical point of view since we observed different stages of AD and compared them with cognitively healthy controls of similar age and cardiovascular status.

One of the limitations of our study was the age difference, the ADD group being slightly older than both other groups. Together with previous findings on age-related alterations of retinal vessel response to flicker, the results of this paper are even emphasized. A progressive reduction of arterial and venous response to flicker was shown in the elderly^42,45^, while an increased vascular response in the slightly older ADD group was observed in the current study. In addition, in post-hoc linear regression analyses age and duration of sleep as potential confounders were forced into the model (data not shown). On the one hand both regression coefficients did not attain statistical significance. On the other hand, all group differences identified in the Mann-Whitney-U-test (Table 3) remained significantly different. Moreover, p-value of difference of mean maximal arterial dilation between MCI and ADD changed from 0.15 to <0.01.

Another limitation concerns the relatively small sample sizes of the groups in the study. Although the high effect sizes allow us to report important findings in retinal neurovascular coupling in AD, further studies are needed to replicate our results in an independent training cohort to confirm the usefulness of dynamic retinal vessel analysis for the diagnosis of AD as compared to established biomarkers.

## Conclusion

Functional retinal arterial and venous reaction to flicker stimulation changed in mild AD dementia with more emphasized arterial and venous dilation as well as delayed arterial reaction. These findings suggest increased and delayed retinal neuro-vascular coupling that may be explained by damaged feed-back loops or abnormally high activity of retinal neurons in AD dementia. In MCI-AD these aspects of retinal vessel behavior were also different from ADD. Further research of vascular dynamic function in AD should reveal the reason for altered behavior of retinal vessels in this disease. Since dynamic retinal vessel analysis provides a direct non-invasive, easy-to-administer and widely-available assessment of retinal vessel behavior and neuro-vascular coupling in the retina, it might offer useful biomarkers for the diagnosis and monitoring of AD in the future.
